# Disability and Comorbidity of Mood Disorders and Anxiety Disorders With Diabetes and Hypertension: Evidences From the China Mental Health Survey and Chronic Disease Surveillance in China

**DOI:** 10.3389/fpsyt.2022.889823

**Published:** 2022-05-20

**Authors:** Yuanyuan Hu, Yueqin Huang, Limin Wang, Zhaorui Liu, Linhong Wang, Jie Yan, Mei Zhang, Ping Lv, Yunqi Guan, Chao Ma, Zhengjing Huang, Tingting Zhang, Hongguang Chen

**Affiliations:** ^1^Peking University Sixth Hospital, Peking University Institute of Mental Health, National Health Commission Key Laboratory of Mental Health (Peking University), National Clinical Research Center for Mental Disorders (Peking University Sixth Hospital), Beijing, China; ^2^National Center for Chronic and Non-communicable Disease Control and Prevention, Chinese Center for Disease Control and Prevention, Beijing, China; ^3^School of Government, Peking University, Beijing, China; ^4^Institute of Social Science Survey, Peking University, Beijing, China

**Keywords:** disability, comorbidity, mood disorder, anxiety disorder, diabetes, hypertension

## Abstract

**Background:**

The China Mental Health Survey was carried out using the same sampling frame with the China Chronic Diseases and Risk Factors Surveillance. This paper explores the relationship between the disability and the comorbidity of mood disorders and anxiety disorders with diabetes and hypertension.

**Methods:**

A large-scale nationally representative sample with both mental disorders and chronic diseases was collected from 157 Disease Surveillance Points in 31 provinces across China. Face-to-face interviews were conducted by trained lay interviewers to make diagnoses of mood disorders and anxiety disorders using the Composite International Diagnostic Interview. Diabetes and hypertension were diagnosed from self-report and blood examination or body measurement. Sampling design weights, non-response adjustment weights, and post-stratification adjustment weights were applied during the analyses of comorbidity and disability.

**Results:**

Totally 15,000 respondents had information of mental disorders and physical diseases. In the patients with mood disorders or anxiety disorders, the weighted prevalence rates of diabetes or hypertension were not higher than those in persons without the above mental disorders, but the weighed disability rates increased when having the comorbidity of hypertension (P < 0.05). The severity of disability was higher among patients with comorbidity of diabetes and anxiety disorders, or hypertension and mood disorders, compared with that among patients without the physical comorbidity (P < 0.05). After adjusted by age, gender and education, patients with comorbidity of mental disorders and physical disorders had the highest disability, followed by the patients with mental disorders only, and physical diseases only.

**Conclusions:**

The disability of mood disorders and anxiety disorders comorbid with diabetes and hypertension are more serious than that of any single disease. The relationship of mental and physical diseases is worth exploring in depth for comprehensive and integrated intervention to decrease the disability.

## Introduction

In 2005 the World Health Organization (WHO) emphasized the importance of mental health in its statement “no health without mental health” (1). It signifies that while under-treated, mental disorders are just as disabling as physical diseases, if not more (2, 3). With the renewed focus on mental health, we start to see that mental disorders and physical diseases do not contribute to morbidity and disability alone: mental disorders increase the risk for physical diseases, and vice versa. Many researchers believe that there exists a bidirectional relationship between mental disorders and physical diseases, with one increasing the odds of onset of the other (4–6), and evidences could be found in many large-sample studies (7–10). The comorbidity of mental disorders and physical diseases tend to make more difficult for patients during the diagnosis and treatment, and therefore influences the prognosis of the diseases (11). Patients with both mental disorders and physical conditions are more likely to have more severe disability than those with any of single conditions (12). The lack of attentions paid to patients with comorbidity may lead to inadequate treatment of mental disorders, poor adherence to the treatment of physical diseases, and lower level of quality of life (8, 13). Disability development may also be prevented by elevating mental health problems (14).

Within mental disorders, mood disorders and anxiety disorders are the most prevalent and play a substantial role in the global burden of disease (11). Findings from the China Mental Health Survey (CMHS) through 2012–2015 in China showed that the 12-month and lifetime prevalence of anxiety disorders were the highest among all mental disorders, and were reported to be 5.0 and 7.6% respectively (15), followed by depressive disorders with 3.6 and 6.8% (16). Within physical diseases, diabetes and hypertension are very common conditions seen in health care facilities, and contribute to many severe subsequent conditions and death if not detected or controlled (17–19). Recent estimates on the prevalence of diabetes and hypertension based on the China Chronic Disease and Risk Factors Surveillance (CCDRFS) study in 2013 were 10.9 (20) and 27.8% (21), respectively. However, China, as a country with the most populous country in the world, has limited evidence on the comorbidity of mental disorders and physical diseases based on a national representative sample. As the CMHS was carried out using the same sampling frame with the CCDRFS (22, 23), it makes possible to carry out a joint analysis of the nationally representative sample collected from 157 Disease Surveillance Points (DSPs) in 31 provinces across China, based on the information of confirmed standard diagnoses for mood disorders, anxiety disorders, diabetes, and hypertension, as well as the status of disability due to the diseases. The aims of this paper are to describe the prevalence and disabilities of comorbid diabetes and hypertension in Chinese adults with existing mood disorders and anxiety disorders, and to explore the relationship between disability and comorbidity.

## Materials and Methods

### Study Design and Study Population

The CCDRFS was conducted in DSPs by the Chinese Center for Disease Control and Prevention (CDC) every 3 years in China. DSPs were originally selected from districts and counties collected by the National Bureau of Statistics based on geographic location, urbanization, economic developmental level, and the population density (24) in each city or country reported from nationwide population census data in 2000. The original DSP system consisted of 161 points was reported with satisfied national representativeness (25). The CCDRFS in 2013 (20, 21) was finally conducted between September 2013 and May 2014 in 298 DSPs, including the original 161 points.

The CMHS (22, 23) was conducted between July 2013 and March 2015 in 157 DSPs selected from the original 161 DSPs. Four DSPs in Tibet were excluded due to difficulties of the fieldwork (23). The CMHS followed the sampling procedures of CCDRFS, but made probability sampling based on the CCDRFS both in communities or villages stage, and household stage. More information of the sampling of the CMHS were described elsewhere (23).

During the field procedure, interviewers of the CMHS finished the household registrations at the same time with the interviewers of the CCDRFS to ensure the same respondent was selected in a household. In order to increase the response rate, a substitution method was used in the CCDRFS if the families could not be accessed three times in 3 days. Instead, an inflated sample size strategy was applied in the CMHS to increase the precision in the estimation of prevalence in the survey. In this paper, the analytic sample was restricted to the respondents who were involved in both the CMHS and CCDRFS.

Because data was managed separately in the CCDRFS and CMHS, matching of the sample was needed when the two surveys were finished. The initial matching was carried out according to the area codes and names of the respondents and verified by gender and age using SAS 9.4. For the remaining of the respondents, two analysts conducted the matching manually based on the Chinese characters of the names. Those respondents having names consisted with similar Chinese characters, with similar age (age difference <3 years old), same area, and same gender were considered as matched sample.

Finally, an overlap sample was then generated. In the sample, all respondents were community residents of Chinese nationality aged 18 years or older who had resided for at least 6 months over the 12 months. Those living in communal residences (workplaces, construction sites, armed services, schools, hospitals, or elderly homes), having problems of hearing, or pregnant women were excluded.

The CCDRFS was approved by the Ethical Review Committee of the Chinese Center for Disease Control and Prevention (201307). The CMHS was approved by the Ethical Committee of the Sixth Hospital of Peking University (IMH-IRB-2013-13-1). All procedures involving human participants were in accordance with the ethical standards of the institutional or national research committee and with the 1964 Helsinki declaration and its later amendments or similar ethical standards. All participants provided their written informed consents for data use. The data of CCDRFS belong to the CDC, while the data of CMHS belong to the National Health Commission of People's Republic of China. All data are not available for sharing.

### Instruments and Measures

In the analytic sample, the diagnoses of mood disorders and anxiety disorders, and the evaluation of disability were collected from the CMHS, and information of diabetes, hypertension, and risk factors was obtained from the CCDRFS.

#### Mental Disorders

The Composite International Diagnostic Interview (CIDI-3.0), a fully structured lay-administered diagnostic interview based on the diagnostic criteria of the Diagnostic and Statistical Manual of Mental Disorders, Fourth Edition (DSM-IV), was applied in the CMHS to made the diagnoses of lifetime mental disorders. Mood disorders (depressive disorder, bipolar disorder, and mood disorder due to a general medical condition) and anxiety disorders (panic attack, agoraphobia without history of panic disorder, specific phobia, social phobia, obsessive compulsive disorder, generalized anxiety disorder, anxiety disorder due to a general medical condition, and anxiety disorder not otherwise specified) were included in this paper. The diagnose of mood or anxiety disorder due to a general medical condition was made by qualified psychiatrists during data cleaning stage. Post-traumatic stress disorder were not included in the analysis as only part of the CMHS sample has the diagnose (22). The reliability and validity of Chinese version CIDI-3.0 had been tested in China (26, 27).

#### Physical Diseases

In this study, the analysis on physical diseases was restricted to diabetes and hypertension, which were included in the CCDRFS with clear diagnoses as well as the information based on self-reports (28).

After an overnight fast of at least 10 h, the blood samples of participants of the CCDRFS were collected for the measurement of fasting plasma glucose and plasma HbA1c. At the same time, they were asked if they had a history of diabetes before the survey, and those who did not have a history of self-reported diabetes were given the oral glucose tolerance test and had their plasma glucose measured at 0 and after 2 hours administration. Total diabetes was the sum of the number of patients who received a diagnosis of diabetes previously determined by a health care professional (self-reported) and the number of patients who had not been diagnosed but blood results had met the diagnostic criteria for diabetes defined according to the American Diabetes Association 2010 criteria, which include fasting plasma glucose level of 126 mg/dL or greater, 2-hour plasma glucose level of 200 mg/dL or greater (after 75 g glucose), or HbA1c concentration of 6.5% or more (20).

Participants of the CCDRFS were asked if they had a history of hypertension before the survey, and had their blood pressure measured three times with at least 1 min interval. Measurement of left upper arm without cloth was preferred. The average of the last two readings was used for analyses. Total hypertension was the sum of the number of patients who received anti-hypertensive treatment in the last 2 weeks, and the number of patients who had not been diagnosed but blood pressure results had met the diagnostic criteria for hypertension defined according to Chinese Guidelines for Prevention and Treatment of Hypertension 2010 criteria, which include mean systolic blood pressure (SBP) ≥140 mm Hg, or mean diastolic blood pressure (DBP) ≥90 mm Hg at survey (21).

#### Functional Disability

The functional disability during the past 30 days was evaluated by the World Health Organization Disability Assessment Schedule 2.0 (WHO-DAS 2.0) (22) in the CMHS. WHO-DAS 2.0 is a 36-item general disability assessment instrument developed by a task force of the WHO, and assesses a variety of impairment and disability dimensions using multi-item scales that reflect six domains of functioning in daily life and allows the comparisons of general disabilities across both mental disorders and physical diseases. Based on the National Classification of Mental Disability (29), mental disability was defined for the patients with mental disorders lasting at least 1 year, and met the criteria of the total score of WHO-DAS 2.0. Three grades of disability were defined as mild (score between 52 and 95), moderate (score between 96 and 105), and severe (score and over).

### Statistical Analysis

The analysis of the association of physical diseases with mental disorders was carried out based on the assumption that mental disorders had earlier onset than that of physical diseases. The rationale for the decision was mainly due to the fact that mental disorders usually develop during adolescence, while physical diseases including diabetes and hypertension tend to occur among middle-aged or older population (30, 31). The decision would decrease the bias during the evaluation of the independent and interactive effects of mental disorders and physical diseases to the disability of the individuals and increase the homogeneity of analytical samples. Only the respondents with consequent diabetes or hypertension after mood disorders or anxiety disorders were included in each analysis. All statistical analyses were performed with SAS 9.4 to adjust for the complex survey design so that the estimates were nationally representative. Weighting was generated based on the weights of the CMHS, but adjusted non-response weights based on the overlap sample. In order to match the population distribution, data was weighted by three steps, including sampling design weights, non-response adjustment weights, and post-stratification adjustment weights. Trimming of the weights was also considered to adjust extreme values (23).

Sociodemographic characteristics were described in percentages of the overall population. Weighted prevalence of physical diseases was calculated among respondents with and without mental disorders. Disability rates and the severity of disability were estimated in patients with mood disorders or anxiety disorders when having the comorbidities of diabetes or hypertension. Standard errors were estimated to determine the 95% confidential interval (CI) for the prevalence or rates using the Taylor series linearization method to adjust for data weighting and clustering. The design-adjusted Rao-Scott χ2 tests were used to examine and the differences of comorbidity rates or disability rates. Logistic regressions were conducted to test the associations between comorbidity and current disability adjusted by age, gender and education level to decrease any report bias due the different understanding of the questions during the interviews. *P* values of < 0.05 using two-sided tests were considered significant.

## Results

### Sample Characteristics

Totally 15,000 respondents in the CMHS were successfully matched the dataset of CCDRFS, with the matching rate of 53.3%. In this sample, 6,535 (43.6%) were males, and 8,465 (56.4%) were females. Totally 1,979 (13.2%) respondents were between 18 and 34 years old, 5,118 (34.1%) respondents were between 35 and 49 years old, 5,520 (36.8%) respondents were between 50 and 64 years old, and 2,383 (15.9%) respondents were 65 years or above.

### Comorbidity of Physical Diseases and Mental Disorders

When comparing the weighted prevalence of physical disorders between respondents with and without mental disorders, there was no difference for the prevalence of diabetes between respondents with and without mental disorders. Patients with mood disorders had lower prevalence of consequent hypertension than those without mood disorders did, while there was no statistical difference of the prevalence of hypertension between respondents with and without anxiety disorders. Detailed results are shown in Table 1.

**Table 1:** Comparison of weighted prevalence of physical diseases between patients with and without mental disorders.

**Conditions**	**With mental disorders**		**Without mental disorders**		*P* ^*^
	**Total**	**% (95%CI)**	**Total**	**% (95%CI)**	
**Mood disorders**					
Diabetes	1,154	10.9	13,734	9.1	0.231
		(7.9–13.9)		(8.1–10.1)	
Hypertension	1,085	20.6	13,668	25.9	0.003
		(16.7–24.5)		(23.5–28.3)	
**Anxiety disorders**					
Diabetes	951	10.5	13,951	9.2	0.344
		(8.0–13.0)		(8.2–10.3)	
Hypertension	937	26.5	13,886	25.6	0.709
		(21.5–31.4)		(23.2–28.0)	

* *Chi test was used to test the difference of prevalence among the two groups*.

The weighted prevalence of diabetes and hypertension among patients with each subtype of mental disorders are shown in Table 2. The respondents with missing data in the diagnoses of physical diseases were excluded in the analysis. The prevalence of comorbid hypertension for most subtype of mental disorders was higher than that of diabetes. Mental disorders due to general medical conditions had the highest prevalence of diabetes or hypertension among all subtypes of disorders. Depressive disorder not otherwise specified (NOS), and anxiety disorder NOS had the lowest prevalence of diabetes among all subtypes of mood disorders or anxiety disorders. Bipolar disorder had the lowest prevalence of hypertension among all subtypes of mood disorders, while obsessive compulsive disorder (OCD) was the lowest among anxiety disorders.

**Table 2:** The weighted prevalence of diabetes or hypertension among patients with each subtype of mental disorders.

**Subtypes of mental disorders**	**Diabetes**		**Hypertension**	
	**Total**	**% (95%CI)**	**Total**	**% (95%CI)**
**Mood disorders**				
Depressive disorders	1,075	10.6 (7.5–13.7)	1012	21.1 (17.0–25.3)
Major depressive disorder	621	11.5 (8.3–14.6)	579	27.4 (22.3–32.4)
Dysthymic disorder	245	12.1 (7.5–16.7)	236	21.2 (13.2–29.2)
Depressive disorder not otherwise specified	427	9.0 (4.6–13.3)	406	15.4 (10.7–20.1)
Bipolar disorder	81	9.2 (1.4–17.1)	78	10.5 (3.9–17.0)
Mood disorder due to general medical conditions	11	51.7 (4.2–99.2)	8	48.0 (7.4–88.6)
**Anxiety disorders**				
Panic attack	95	13.6 (2.8–24.4)	94	22.6 (7.4–37.9)
Agoraphobia without history of panic disorder	66	14.0 (0.4–27.6)	65	29.0 (12.2–45.7)
Specific phobia	471	12.3 (7.7–16.9)	466	28.6 (22.2–35.0)
Social phobia	111	10.3 (2.2–18.4)	110	31.4 (17.8–45.1)
Obsessive compulsive disorder	345	9.2 (4.7–13.6)	336	21.5 (14.8–28.3)
Generalized anxiety disorder	58	14.6 (2.9–26.4)	58	35.9 (20.3–51.6)
Anxiety disorder due to general medical conditions	19	45.2 (4.6–85.7)	19	78.0 (62.2–93.9)
Anxiety disorder not otherwise specified	110	9.1 (2.5–15.7)	108	30.0 (17.6–42.5)

### The Disability Rate and Severity of Disability Among Patients With Mental Disorders Across Different Comorbidities

Table 3 shows the comparisons of the weighted disability rates among patients with mental disorders across the status of diabetes or hypertension. Only patients with the comorbidity of mental disorders and hypertension had higher disability rate than the patients without hypertension did. There was no statistical difference of the weighted disability rates between other pairs. Table 3 also further explores the differences of the severity of disability across the comorbidity. Among all disabled patients with mood disorders, only patients with the comorbidity of hypertension had higher level of disability than that among patients without the comorbidity. Among all disabled patients with anxiety disorders, patients with the comorbidity of diabetes had more severe disability compared with those without the comorbidity did.

**Table 3:** Comparison of the weighted disability and the severity of disability across the status of physical diseases.

**Conditions**	**With physical diseases**		**Without physical diseases**		P^^*^^
	***N***	**% (95%CI)**	***N***	**% (95%CI)**	
**Diabetes**					
Mood disorders	76	42.5 (29.9–55.1)	393	34.2 (29.8–38.5)	0.218
Severe	16	17.6 (5.2–30.0)	36	10.2 (5.8–14.7)	
Moderate	4	3.0 (0.0–7.0)	25	4.5 (1.4–7.6)	0.339
Mild	56	79.4 (66.2–92.6)	332	85.3 (79.7–90.8)	
Anxiety disorders	54	41.7 (29.0–54.3)	318	34.9 (29.8–40.0)	0.319
Severe	8	12.9 (0.3–25.5)	26	6.9 (4.9–9.0)	
Moderate	4	21.1 (0.0–45.5)	14	3.9 (0.5–7.2)	0.005
Mild	42	66.0 (43.0–89.1)	278	89.2 (84.8–93.6)	
**Hypertension**					
Mood disorders	148	44.0 (33.9–54.2)	281	31.1 (26.2–36.1)	0.028
Severe	23	18.7 (5.9–31.6)	23	7.0 (3.5–10.5)	
Moderate	8	4.2 (0.5–7.9)	16	4.7 (1.0–8.3)	0.018
Mild	117	77.0 (65.3–88.7)	242	88.3 (83.0–93.6)	
Anxiety disorders	152	51.4 (43.1–59.7)	212	29.8 (24.2–35.4)	<0.001
Severe	23	13.5 (9.2–17.9)	10	4.2 (1.4–7.0)	
Moderate	6	4.1 (0.0–10.7)	12	7.3 (1.2–13.4)	0.089
Mild	123	82.4 (75.2–89.6)	190	88.5 (81.3–95.7)	

* *Chi test was used to test the difference of prevalence among the two groups*.

The weighted disability rates and the severities of disability among patients with each subtype of mental disorders across different comorbidity status are presented in Table 4. The disability rates among patients with most subtypes of mood disorders were similar between the two types of comorbidities, except for patients with dysthymic disorder and mood disorder due to general medical conditions. The disability rates among patients with some subtypes of anxiety disorders were similar between diabetes and hypertension. But having comorbidity of hypertension among patients with panic attack, social phobia, OCD, and anxiety disorder NOS had higher disability rates than those with diabetes.

**Table 4:** Weighted disability rates and the severity of disability among patients with each subtype of mental disorders across different comorbidity status.

**Subtype of mental disorders**	**Diabetes**					**Hypertension**				
	**Total**	**Disability rate**	**Severity of disability (%)**			**Total**	**Disability rate**	**Severity of disability (%)**		
		**% (95%CI)**	**Severe**	**Moderate**	**Mild**		**% (95%CI)**	**Severe**	**Moderate**	**Mild**
**Mood disorders**											
Depressive disorders	141	39.3 (25.1–53.5)	14.6	2.3	83.1	300	43.6 (33.1–54.1)	18.3	4.4	77.3
Major depressive disorder	95	48.9 (33.0–64.8)	13.5	0.8	85.7	191	52.6 (36.9–68.3)	16.2	5.0	78.8
Dysthymic disorder	42	29.4 (9.7–49.1)	9.6	2.9	87.5	75	55.0 (36.0–73.9)	28.4	9.8	61.8
Depressive disorder not otherwise specified	41	33.4 (12.6–54.3)	16.6	4.9	78.4	99	29.3 (15.0–43.7)	25.9	3.3	70.7
Bipolar disorder	11	58.4 (18.8–97.9)	0.0	0.0	100.0	20	59.3 (31.3–87.2)	17.7	0.0	82.3
Mood disorder due to general medical conditions	4	100.0 (100.0–100.0)	60.0	13.0	27.0	4	21.0 (0.0–57.9)	85.0	0.0	15.0
**Anxiety disorders**											
Panic attack	9	32.3 (0.0–75.5)	0.0	0.0	100.0	27	56.5 (22.4–90.5)	11.9	3.1	84.9
Agoraphobia without history of panic disorder	12	75.1 (34.7–100.0)	0.0	62.5	37.5	24	73.6 (45.4–100.0)	6.7	31.0	62.3
Specific phobia	65	45.3 (31.0–59.6)	5.6	35.8	58.6	177	43.9 (33.2–54.5)	10.2	8.5	81.3
Social phobia	14	56.0 (15.0–97.1)	0.0	62.7	37.3	39	71.6 (46.4–96.9)	20.3	17.1	62.6
Obsessive compulsive disorder	34	27.5 (9.7–45.3)	11.9	0.0	88.1	103	52.4 (37.6–67.2)	7.6	0.0	92.4
Generalized anxiety disorder	9	74.8 (32.6–100.0)	4.7	0.0	95.3	20	71.7 (44.8–98.6)	19.6	2.4	77.9
Anxiety disorder due to general medical conditions	7	100.0 (100.0–100.0)	53.5	0.0	46.5	14	100.0 (100.0–100.0)	62.0	0.0	38.0
Anxiety disorder not otherwise specified	12	23.8 (0.0–48.1)	46.7	0.0	53.3	38	39.4 (16.8–62.0)	10.7	0.0	89.3

For the comorbidity of diabetes, patients with mood disorder or anxiety disorder due to general medical conditions had the highest disability rate among all subtypes of mood disorders or anxiety disorders, while patients with dysthymic disorder and anxiety disorder NOS had the lowest disability rate. For the comorbidity of hypertension, patients with bipolar disorder and anxiety disorder due to general medical conditions had the highest disability rate, while patients with mood disorder due to general medical conditions and anxiety disorder NOS had the lowest disability rate.

Further analyses for the severity of disability showed the severity levels of disability among most subtype of mood disorders and anxiety disorders were more severe among patients with the comorbidity of hypertension compared with those with diabetes, except for OCD and anxiety NOS. Patients with mental disorders due to general medical conditions had the highest level of severity among all subtypes of mood disorders or anxiety disorders. Patients with the comorbidity of bipolar disorder and diabetes or hypertension had the lowest level of severity among all subtypes of mood disorders. Patients with the comorbidity of panic attack and diabetes had the lowest level of severity among all subtypes of anxiety disorders, while patients with the comorbidity of OCD and hypertension had the lowest level of severity among anxiety disorders.

### The Association of Comorbidity With Current Disability

Findings from the logistic regression models to test the association of comorbidity with disability are shown in Table 5. Compared with the reference group (those without mental disorder or physical disease), the odds ratios (ORs) of the comorbidity with mental disorders and physical diseases were the highest among all variables, followed by the ORs of having mental disorders only. Having physical diseases only had the smallest contribution to the disability.

**Table 5:** The association of comorbidity with current disability^*^.

**Variables**	**Diabetes**			**Hypertension**		
	**Wald** **χ^2^**	**OR (95% CI)**	**P**	**Wald** **χ^2^**	**OR (95% CI)**	***P***
**Mood disorders**							
Without mental disorder or physical disease		1			1	
Physical diseases only	5.19	1.21 (1.03–1.42)	0.023	12.88	1.25 (1.11–1.41)	<0.001
Mental disorders only	618.17	11.91 (9.80–14.48)	<0.001	492.74	12.88 (10.28–16.14)	<0.001
Physical diseases and mental disorders	109.04	13.41 (8.24–21.83)	<0.001	230.00	13.36 (9.56–18.68)	<0.001
**Anxiety disorders**							
Without mental disorder or physical disease		1			1	
Physical diseases only	5.93	1.22 (1.04–1.43)	0.015	9.68	1.21 (1.07–1.37)	0.002
Mental disorders only	500.55	8.57 (7.10–10.35)	<0.001	361.78	8.72 (6.98–10.91)	<0.001
Physical diseases and mental disorders	96.39	10.46 (6.55–16.71)	<0.001	247.31	9.86 (7.41–13.11)	<0.001
**Mood or anxiety disorders**							
Without mental disorder or physical disease		1			1	
Physical diseases only	5.87	1.23 (1.04–1.46)	0.015	11.29	1.25 (1.10–1.42)	0.001
Mental disorders only	810.16	9.70 (8.29–11.34)	<0.001	598.56	10.19 (8.46–12.27)	<0.001
Physical diseases and mental disorders	155.33	10.89 (7.48–15.86)	<0.001	347.96	10.41 (8.14–13.32)	<0.001

*Adjusted by age, gender and education.

## Discussion

The study explores evidence of comorbidities of mental disorders and physical diseases in the most populous country in the world, providing an important opportunity for understanding the global disease burden. The main contributions of the nationwide mental-physical study are to report the prevalence rates of comorbidities of mood disorders and anxiety disorders with diabetes and hypertension, as well as the disability rates and the severities of disabilities for most subtypes of mood disorders and anxiety disorders. Findings from the survey support the general argument that the comorbidity of physical diseases and mental disorders would increase the disability rate and the severity of disability (12). However, this study indicated that the disability varied in different status of comorbidity. Hypertension increased the disability rate of patients with mood and anxiety disorders, but that was not found for diabetes. Hypertension only increased the severity of disability for patients with mood disorders, while diabetes only increased the severity of disability for patients with anxiety disorders. The disability rates and the severities of disability varied in different subtypes of mood disorders and anxiety disorders. These findings imply that different strategies should be applied to decrease the disability of mood or anxiety disorders when patients having the comorbidities of physical diseases.

Most previous studies on comorbidities of mental disorders and physical diseases relied on self-reported data (32), or were based on patients with physical diseases (2, 33). Some studies about the comorbidity of diabetes and hypertension in patients with mood disorders and anxiety disorders reported lower (34) or higher (35) rates than the current finding. The most likely reasons for the discrepancy were the lack of representativeness of populations, valid diagnoses of mental disorders, and self-reported diagnoses of diabetes or hypertension through questionnaire instead of accurate examination such as structured CIDI-3.0 for mental disorders, body measurements for blood pressure, and blood tests for blood glucose. This study explores the significance of psychosomatic diseases in the Chinese population from the perspective of mental disorders, which also makes this research unique and innovative.

In the current study, the weighted prevalence of diabetes or hypertension among patients with mental disorders varied in the subtypes of mood or anxiety disorders, but the overall prevalence was not higher than that in persons without mental disorder, and the weighted prevalence of hypertension in respondents with mood disorders (20.6%) was even significantly lower than the prevalence among respondents without mood disorder (25.9%). As this study analyzed the relationship between mental disorders and consequent physical diseases, the above findings indicate that mental disorders might not the cause of the increase of physical disorders, such as hypertension or diabetes, which is different with the conclusion made in a previous large sample survey conducted in 17 countries (7). The discrepancy might due to the difference of the population, or the measurements of hypertension or diabetes.

It is worth noting that among the sub-categorical mood disorders and anxiety disorders, patients with mental disorder due to general medical conditions usually had the highest disability rate and most severe disability when having the comorbidity of physical diseases compared with other subtypes of mood or anxiety disorders did. It implies that mental and physical conditions are harmonious under integration of body and mind with interaction. It should be paid attention for physicians and psychiatrists to provide concurrent treatment for mental disorders and physical diseases.

The coexistence of mental-physical disease makes the condition of disease more serious, complicated and costly (36), which not only affects physical health but also functional decline, even causing disability with the potential for a major epidemic of complications (3). Despite considerable evidence that mental disorders and physical diseases were responsible for reducing daily functions and increasing the risk of disabilities, little is done for psychiatrists to diagnose or treat physical illness for patients with mental disorders (37). More training should be provided for psychiatrists to deal with physical illness in patients with mental disorders. Furthermore, physicians should pay attention to mental symptoms during general practice. Liaison psychiatry is an important field in order to treat physical and mental disorders.

This study was one part of CMHS and CDRFS in 2013, the first nationwide cross-sectional study on mental disorders integrated into physical diseases. Both mental and physical symptoms were collected at same time so that comorbidities of mental disorders with diabetes and hypertension could be analyzed. This is an unprecedented nationally representative epidemiological study on mental and physical health in the world. The study frame represented up to approximately 1.09 billion Chinese adult population aged 18 years or older due to multiple stage sampling on 157 DSPs, which was used to ensure this study countrywide representative in the year of 2013 (38). In the current study, CIDI-3.0 which is a validated fully structured diagnostic interview was used to diagnose mood disorders and anxiety disorders. At same time, diabetes diagnosis was obtained by previously physician diagnosed as well as three glycemic indexes by blood samples test according to the American Diabetes Association 2010 criteria, and hypertension diagnosis was obtained by previously physician diagnosed as well as blood pressure indexes according to Chinese Guidelines for Prevention and Treatment of Hypertension 2010 criteria. Therefore, the standardization of diagnoses made the results of this study more valid and reliable.

This study had some limitations. First, the onsets of mental disorders and physical diseases were estimated based on self-report, rather than medical records. The recall bias could not be avoided. Second, treatment histories of physical diseases were not collected in the survey, which might influence the estimation of the disability and the diagnoses of diabetes or hypertension. Third, the comorbidity of having both diabetes and hypertensions was not considered during the analysis. Forth, about half of the respondents in the CMHS had information of the diagnoses of hypertension or diabetes. The comorbidity of the rest of sample was not clear.

## Conclusion

From this nationwide representative epidemiological survey in China, it shows that the disabilities of mood disorders and anxiety disorders comorbid with diabetes and hypertension are higher than that of any single disease. Considering the importance of precise treatment and prevention, this current finding may enhance the assessment and intervention methods tailored differently to different comorbidities.

## Data Availability Statement

The datasets presented in this article are not readily available because the corresponding authors had full access to all the data collected in the survey. Only receiving permission from relevant organization shall the data be shared with other researchers. Requests to access the datasets should be directed to YHua, huangyq@bjmu.edu.cn.

## Ethics Statement

The CCDRFS was approved by the Ethical Review Committee of the Chinese Center for Disease Control and Prevention (201307). The CMHS was approved by the Ethical Committee of the Sixth Hospital of Peking University (IMH-IRB-2013-13-1). The participants provided their written informed consent to participate in this study.

## Author Contributions

YHua and LinW originally designed the study and have been responsible for obtaining funding. They also contributed to the study design, development of study instruments, field work, and data analysis. ZL and YHu wrote the first draft. YHu undertook data cleaning, checking and coding, and did the analysis with the help from CM. LimW and JY led project management. MZ, PL, YG, ZH, TZ, and HC participated in data collecting and analyzing. YHua, LimW and ZL conceived the idea for this manuscript, supervised and checked the analysis, and the final draft. All authors contributed to this study, read the manuscript, approved the final manuscript, interpretation of data, and the approval of the final report.

## Funding

This study was supported by the National Key R&D Program of China (Nos. 2017YFC0907800 and 2017YFC0907801), the National Twelfth Five-year Plan for Science and Technology Support of the Ministry of Science and Technology (Nos. 2015BAI13B01 and 2012BAI01B00), the Special Research Project for Non-profit Public Service of Ministry of Health (No. 201202022), the Chinese Central Government (key project of public health program), and the National Natural Science Foundation of China (Nos. 81473043, 81230066, and 91546120).

## Conflict of Interest

The authors declare that the research was conducted in the absence of any commercial or financial relationships that could be construed as a potential conflict of interest.

## Publisher's Note

All claims expressed in this article are solely those of the authors and do not necessarily represent those of their affiliated organizations, or those of the publisher, the editors and the reviewers. Any product that may be evaluated in this article, or claim that may be made by its manufacturer, is not guaranteed or endorsed by the publisher.
